# Inhibitory Mechanisms of DHA/CQ on pH and Iron Homeostasis of Erythrocytic Stage Growth of *Plasmodium falciparum*

**DOI:** 10.3390/molecules24101941

**Published:** 2019-05-20

**Authors:** Tian Tang, Wenhui Xu, Ji Ma, Huajing Wang, Zhao Cui, Tingliang Jiang, Canghai Li

**Affiliations:** 1 Tang Center for Herbal Medicine Research, China Academy of Traditional Chinese Medical Sciences, Beijing 100700, China; tinatang17@163.com (T.T.); xwh1428@126.com (W.X.); mj890.com@163.com (J.M.); Whjdd91@163.com (H.W.); cuizhao1124@126.com (Z.C.); tljiang@icmm.ac.cn (T.J.); 2 Artemisinin Research Center and Institute of Chinese Meteria Medica, China Academy of Traditional Chinese Medical Sciences, Beijing 100700, China

**Keywords:** malaria, drug combinations, dihydroartemisinin, chloroquine, baflomycin-A1, V-type proton ATPase inhibitor, *Plasmodium falciparum*, digestive vacuole, calcein, BCECF

## Abstract

Malaria is an infectious disease caused by *Plasmodium* group. The mechanisms of antimalarial drugs DHA/CQ are still unclear today. The inhibitory effects (IC_50_) of single treatments with DHA/CQ or V-ATPase inhibitor Baf-A1 or combination treatments by DHA/CQ combined with Baf-A1 on the growth of *Plasmodium falciparum* strain 3D7 was investigated. Intracellular cytoplasmic pH and labile iron pool (LIP) were labeled by pH probe BCECF, AM and iron probe calcein, AM, the fluorescence of the probes was measured by FCM. The effects of low doses of DHA (0.2 nM, 0.4 nM, 0.8 nM) on gene expression of V-ATPases (vapE, vapA, vapG) located in the membrane of DV were tested by RT-qPCR. DHA combined with Baf-A1 showed a synergism effect (CI = 0.524) on the parasite growth in the concentration of IC_50_. Intracellular pH and irons were effected significantly by different doses of DHA/Baf-A1. Intracellular pH was decreased by CQ combined with Baf-A1 in the concentration of IC_50_. Intracellular LIP was increased by DHA combined with Baf-A1 in the concentration of 20 IC_50_. The expression of gene vapA was down-regulated by all low doses of DHA (0.2/0.4/0.8 nM) significantly (*p* < 0.001) and the expression of vapG/vapE were up-regulated by 0.8 nM DHA significantly (*p* < 0.001). Interacting with ferrous irons, affecting the DV membrane proton pumping and acidic pH or cytoplasmic irons homeostasis may be the antimalarial mechanism of DHA while CQ showed an effect on cytoplasmic pH of parasite in vitro. Lastly, this article provides us preliminary results and a new idea for antimalarial drugs combination and new potential antimalarial combination therapies.

## 1. Introduction

Malaria is a mosquito-borne infectious disease affecting humans and other animals caused by single-celled microorganisms belonging to the *Plasmodium* group. There were estimated 219 million clinical cases of malaria and 435,000 deaths from malaria globally in 2017. *Plasmodium falciparum* (*P. f*) is the most prevalent malaria parasite in most of the WHO Regions [1]. Chloroquine (CQ), one of the World Health Organization’s List of Essential Medicines, has long been used in the treatment or prevention of malaria, while *P. falciparum* has developed widespread resistance to it. Artemisinins (ARTs) in combination with other antimalarial drugs (artemisinin-combination therapies/ACTs) have been integral to the recent success of global malaria control, especially for *P. falciparum*. Dihydroartemisinin (DHA) is the active metabolite and widely used as an intermediate of all artemisinin-derived compounds (artemisinin; artesunate; artemether; etc.). It is also used in combination with piperaquine to treat malaria generally.

Many studies have showed that the de novo heme biosynthesis process by malaria parasites to satisfy the critical metabolic needs is thought to be an important potential target for antimalarial drugs development [2]. However, whether these antimalarial drugs interference with heme or hemozoin (Hz) digested from hemoglobin (Hb) in the parasite digestive vacuole, is still under discussion by many researchers.

Accumulation inside the acidic digestive vacuole (DV or food vacuole) due to its alkalinity through an ion trapping mechanism [3], interference with the process of heme accumulation and Hz formation, and interference of membrane integrity are reported to be the mechanism of CQ [4,5]. Upon entering the acidic environment, CQ is doubly protonated and becomes membrane impermeable, causing it to reach millimolar concentration levels compared to nanomolar concentration levels in the plasma [6]. CQ resistance in *P. falciparum* has been found to be associated with mutations in a parasite DV membrane protein, *P. falciparum* CRT (Pf CRT) [7]. Meanwhile, CQ is an inhibitor of lysosomal protein degradation to induce cell death, an endosomal acidification inhibitor which inhibits lysosomal enzymes that require an acidic pH and prevents fusion of endosomes and lysosomes [8,9,10,11].

The exact mechanism of the antimalarial ART family compounds is highly debated, it is generally agreed that the endoperoxide bridge of ART and its derivatives is activated by iron, causing free radicals and reactive oxygen species (ROS) to form inside the parasite [12]. The ferrous irons present in the host-derived heme are the major catalysts and activators that mediate the breakdown of the endoperoxide bridge. Studies have shown that heme may be an electron donor during the activation process, but the target site or organelle of DHA and its activation mechanism are still uncertain. It has been reported that inhibition of Hb digestion decreased the sensitivity of artemisinin to malaria parasites [13], suggesting that hemoglobin-derived heme plays an important role in activation with DHA. Within heme derived from Hb and leakage from the DV into the cytoplasm of malaria parasites, ferrous irons (Fe^2+^) are more conducive to the activation of artemisinins [14]. We hypothesize that DHA is activated by ferrous irons or high concentration levels of heme in DV from Hb digestive process, which may be a different antimalarial mechanism from CQ.

During the lifecycle within a human (or other mammalian) red blood cell (RBC), *P. falciparum* digests the host erythrocyte Hb, using it as a source of amino acid (AA) in the DV for growth and reproduction. DV, a membrane-bound organelle, is the critical organelle containing hydrolytic enzymes and other proteins of Hb digestion and the formation of the large hemozoin crystals [15]. In blood stages, malaria parasites consume most of the Hb inside the infected erythrocytes, forming nontoxic Hz crystals from large quantities of heme released during the process of Hb digestion. The growth and reproduction of malaria parasites acquires AA and stores irons as Hz to prevent free heme toxicity. Heme metabolism is central to malaria parasite biology. Malaria parasites possess a de novo heme biosynthetic pathway, which is considered to be essential and is proposed as a potential drug target. Hangjun Ke demonstrate that the de novo heme biosynthesis pathway is not essential for asexual blood-stage growth of *P. falciparum* parasites but is required for mosquito stages [2].

DV maintains its differential pH (4.8–5.2) by pumping in protons from the cytosol across the membrane via proton pumps or chloride ion channels, this acidic environment gives service to the accumulation of detoxified heme and massive ferrous irons during the hydrolization of Hb in the DV [16,17]. Transporters, located in the membrane of DV, such as vacuolar iron transporter (VIT), sodium hydrogen exchanger (NHE), chloroquine resistance transporter (CRT), responding for drug and maintaining a steady acidic environment, are reported to be responsible for transmembrane transport and balance of ions in DV. Vacuolar-type ATPases (V-ATPases) coupling the energy of ATP hydrolysis to proton transport across intracellular and plasma membranes of parasites play a crucial role in ATP hydrolysis coupled proton transmembrane transport, vacuolar acidification, pH homeostasis, ATP binding and ATP metabolic process [18]. V-ATPases, found within the membrane of DV, are responsible for proton pumping into DV and the acidification process of DV. While other V-ATPases, found within the plasma membranes, are responsible for excretion of proton from intracellular.

Bafilomycin A1 (Baf-A1) is a selective inhibitor of V-ATPases and has an inhibitory effect on vacuolar ATPases, which can be further determined by testing its influence on proton pumping activity. Baf-A1 is also known as an inhibitor of the late phase of autophagy for preventing the maturation of autophagic vacuoles by inhibiting fusion between autophagosomes and lysosomes [19]. Baf-A1 is a member of a potent family of macrocyclic lactones with broad spectrum biological activity, including activity against bacteria, yeast, fungi, nematodes, insects and tumour cell lines.

The mechanisms of most current antimalarial drugs, for example: CQ, DHA or other artemisinins, still remain unclear over these years. New antimalarial combination therapies or new drugs are to be the urgent need in the battle with malaria all over the world, for these frontier antimalarial drugs have developed drug resistance or multidrug resistance during these years. Studies for the mechanism of antimalarial drugs or combination drugs can meet the demand for resolving of the resistance problem. So it’s considered to supply a new thought of combination treatment by V-ATPase inhibitor with different antimalarial drugs in this in vitro study.

**Figure 1:**
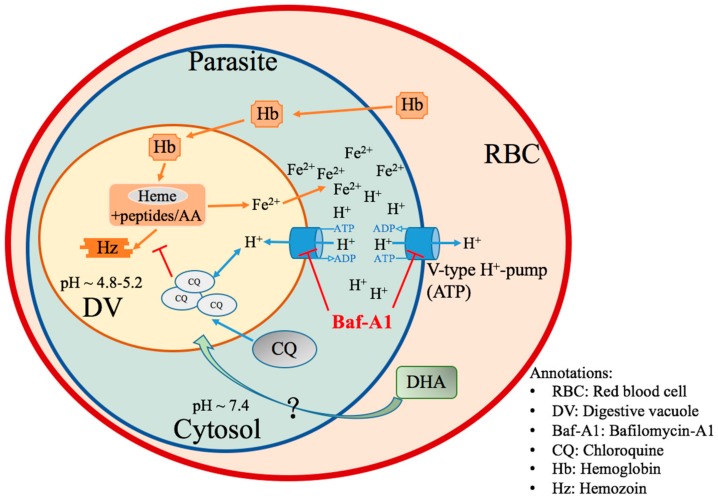
The mechanisms of antimalarial drugs chloroquine (CQ)/dihydroartemisinin (DHA) and V-ATPase inhibitor Baf-A1 on malaria parasites.

Accordingly, the mechanisms of current antimalarial drugs DHA/CQ and V-ATPase inhibitor Baf-A1 on malaria parasites, as seen in Figure 1, may be close to the ions hemostasis of parasites during their lifecycle. Their targets, as seen in Table 1, may be mainly located at DV or in the membrane. We focused our study points on the effects of these drugs when dealing with malaria parasite, *Plasmodium falciparum* laboratory strain 3D7, a susceptible strain of drug not the multidrug-resistant strain.

## 2. Results

### 2.1. Single Drug Treatment and Combination Drug Treatment Testing

#### 2.1.1. Single Drug Treatment to Assay IC_50_

To explore the concentration of drug combination treatment, we tested the IC_50_ values by single drug treatment of DHA, CQ and Baf-A1 against *P. falciparum* strain 3D7. The stage-specific IC_50_ of drugs on *P. f* 3D7 blood-stage cultures is shown in Table 2. The lower IC_50_ values represent the higher inhibitory effects of drugs on parasite growth. The IC_50_ value of DHA is 4.12 nM. DHA is more effective than CQ to inhibit the growth of *P. f* 3D7 in vitro. The growth of malaria parasites is also inhibited by V-ATPase inhibitor Baf-A1 (IC_50_ = 25.15 nM) in a low nanomolar concentration, and the IC_50_ value of Baf-A1 is approaching to that of CQ (IC_50_ = 18.74 nM).

**Figure 2:**
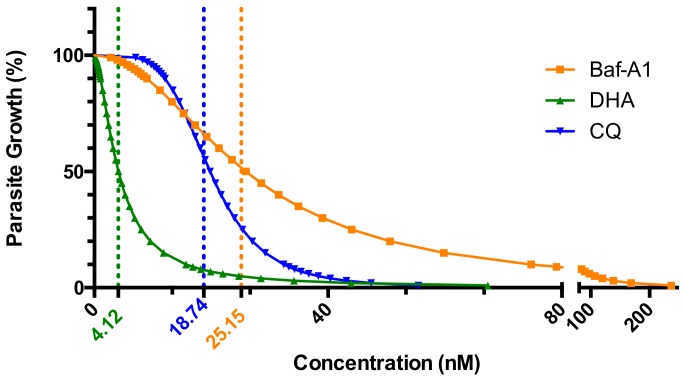
Survival curves of *P. f* 3D7 treated with Baf-A1 and anti-malarial drugs CQ/DHA. Data was obtained from three independent triplicate experiments. Their IC_50_ values were shown in dotted lines.

Survival curves of *P. falciparum* strain 3D7 treated with Baf-A1 and antimalarial drugs CQ/DHA were shown in Figure 2. The survival curves of malaria parasites treated by DHA/CQ were similar with S-curve, while that of Baf-A1 was different from DHA/CQ.

#### 2.1.2. Combination Drug Treatment to Assay Combination Effects

Antimalarial drugs combination treatment with Baf-A1 were tested at a concentration level of IC_50_ and a series of ratio of IC_50_. Combination index (CI) values of DHA/CQ with Baf-A1 in serial doses against *P. falciparum* strain 3D7 were shown in Table 3 and Figure 2.

**Figure 3:**
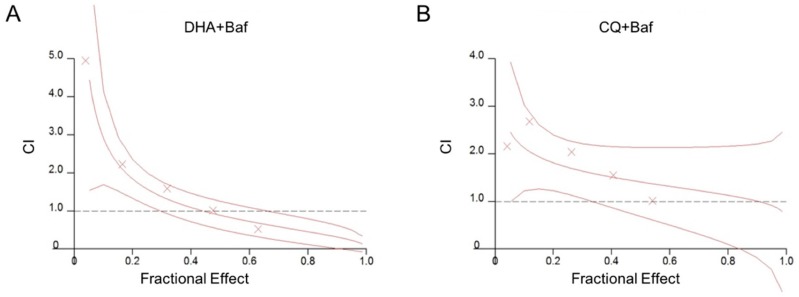
Effects of the antimalarial drugs DHA/CQ combined with Baf-A1 against *P. f* 3D7 growth in vitro. (**A**) CI of DHA combined with Baf-A1, (**B**) CI of CQ combined with Baf-A1, combination index (CI) represents the interaction effect in the presence and absence of inhibitor. CI < 1, =1, and >1 indicates synergism, additive, and antagonism effect, respectively.

Results of combination drugs DHA/CQ with Baf-A1 on the parasite growth inhibition are shown in Table 3 and Figure 3, all the antimalarial drugs combination treatment with Baf-A1 at low concentrations showed antagonism effect (CI > 1), DHA combined with Baf-A1 at a concentration of 20% IC_50_ showed strong antagonism effect (CI = 4.954). DHA combined with Baf-A1 at a concentration of IC_50_ showed synergism effect (CI = 0.524), while CQ combined with Baf-A1 showed only antagonism effect or nearly additive effect (CI = 1.012) in all groups.

### 2.2. Cellular pH Test by Flow Cytometry (FCM) Using pH Probe BCECF, AM

#### 2.2.1. Single Drug Treatment

The intracellular pH ranging from 4 to 9 was indicated by the fluorescence intensity of BCECF (Em = 535 nm, Ex = 488 nm), low intensity indicates acid pH and heightened intensity of BCECF represent a shift toward alkaline pH.

**Figure 4:**
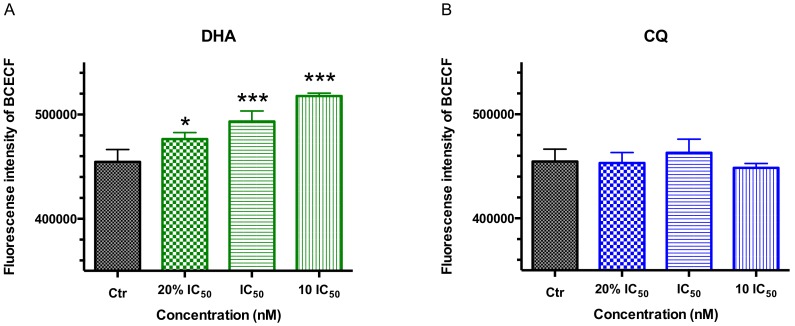

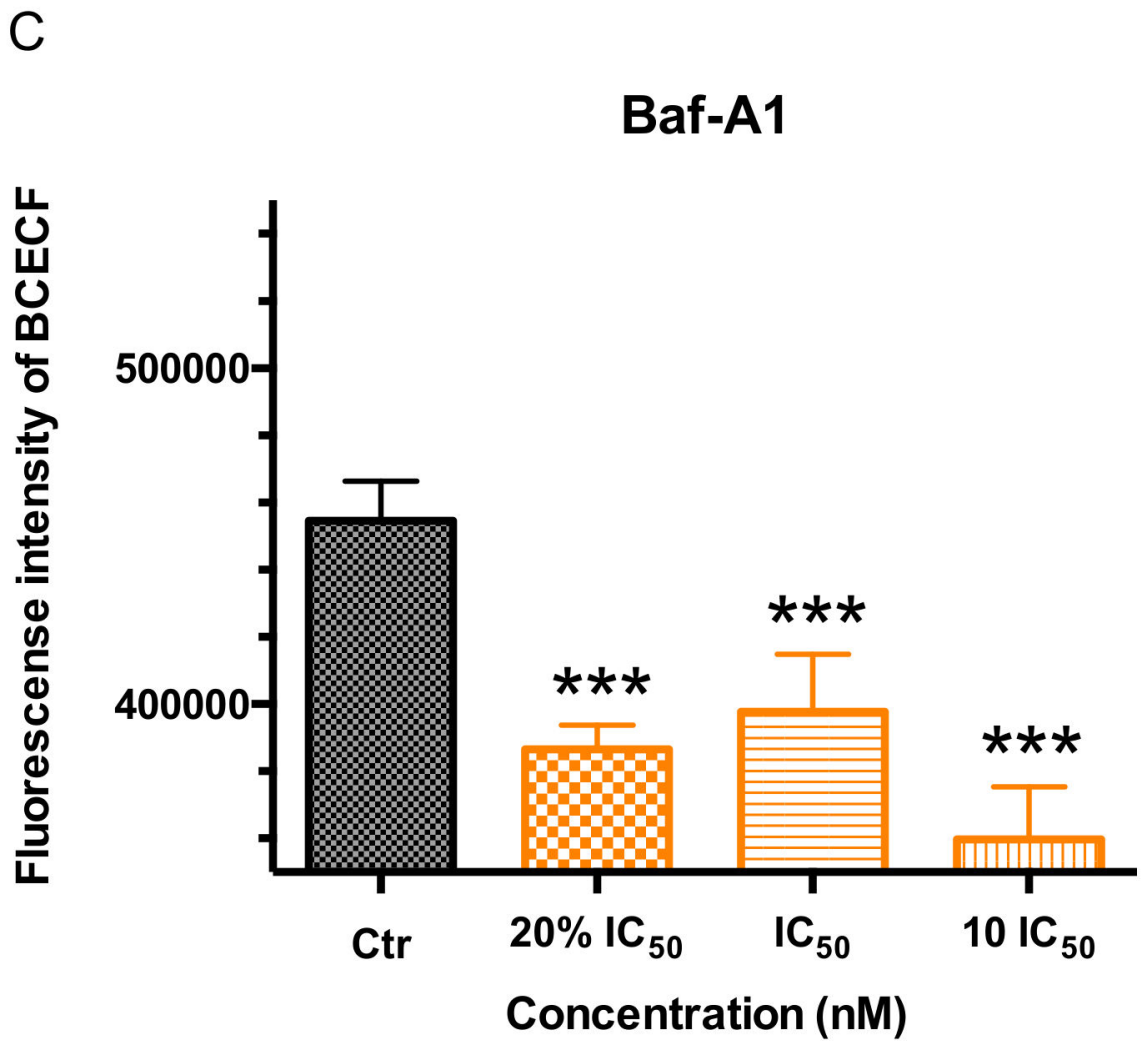
The fluorescence intensity of parasites in trophozoite stage *P. f* 3D7 staining with pH probe BCECF, AM after 2 h of single drug treatment with a series dose of antimalarial drugs DHA /CQ or V-ATPase inhibitor Baf-A1 by flow cytometry. (**A**) Fluorescence intensity of BCECF in trophozoite stage *P. f* 3D7 treated by DHA; (**B**) Fluorescence intensity of BCECF in trophozoite stage *P. f* 3D7 treated by CQ; (**C**) Fluorescence intensity of BCECF in trophozoite stage *P. f* 3D7 treated by Baf-A1. Ctr means control. Data shown are mean ± SEM (n ≥ 3). * *p* < 0.05 or *** *p* < 0.001 vs. control.

The fluorescence intensity of parasites staining with pH probe BCECF, AM after 2 h of single drug treatment with a series dose of antimalarial drugs DHA/CQ or V-ATPase inhibitor Baf-A1 by flow cytometry was shown in Figure 4. All of the DHA groups (10 IC_50_/IC_50_/20% IC_50_) increased the fluorescence intensity of BCECF and intracellular pH of parasite significantly with a dose-effect relationship, while the fluorescence intensity of BCECF decreased significantly when treated by all doses of Baf-A1 (*p* < 0.001).

#### 2.2.2. Combination Drug Treatment Testing

**Figure 5:**
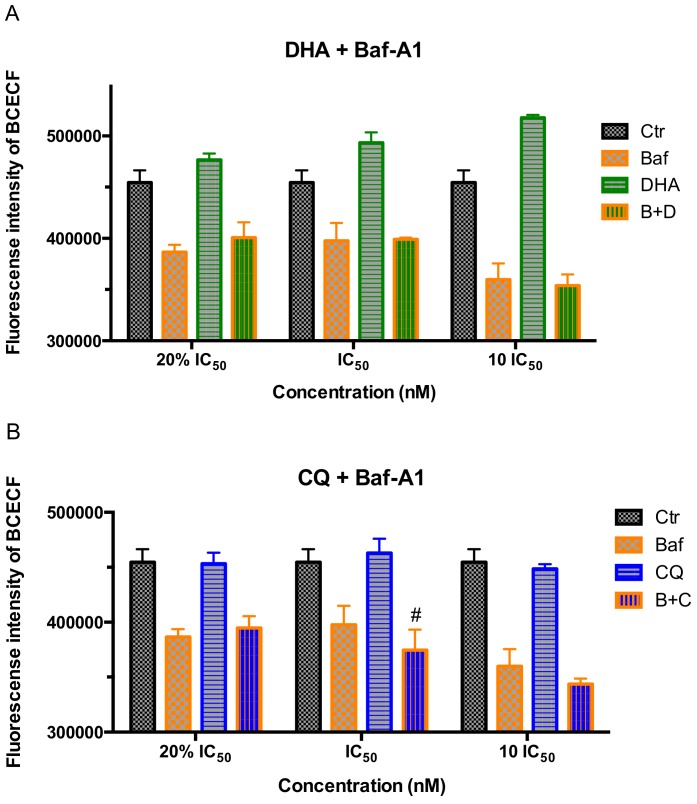
The fluorescence intensity of parasites in trophozoite stage *P. f* 3D7 staining with pH probe BCECF, AM after 2 h of combination drug treatment with a series dose of antimalarial drugs DHA/CQ and V-ATPase inhibitor Baf-A1 by flow cytometry. (**A**) Fluorescence intensity of BCECF in trophozoite stage *P. f* 3D7 treated by DHA combined with Baf-A1; (**B**) Fluorescence intensity of BCECF in trophozoite stage *P. f* 3D7 treated by CQ combined with Baf-A1. Ctr means control, B+D means DHA combined with Baf-A1, B+C means CQ combined with Baf-A1. Data shown are mean ± SEM (n ≥ 3). # *p* < 0.05 vs. Baf-A1.

The fluorescence intensity of parasites staining with pH probe BCECF, AM after 2 h of combination drug treatment with a series dose of antimalarial drugs DHA/CQ and V-ATPase inhibitor Baf-A1 by flow cytometry was shown in Figure 5. The fluorescence intensity of BCECF was decreased by CQ combined with Baf-A1 in the concentration of IC_50_ significantly when compared with Baf-A1 (*p* < 0.05).

### 2.3. Labile Iron Pool Test by Flow Cytometry Using Probe Calcein, AM

#### 2.3.1. Single Drug Treatment

**Figure 6:**
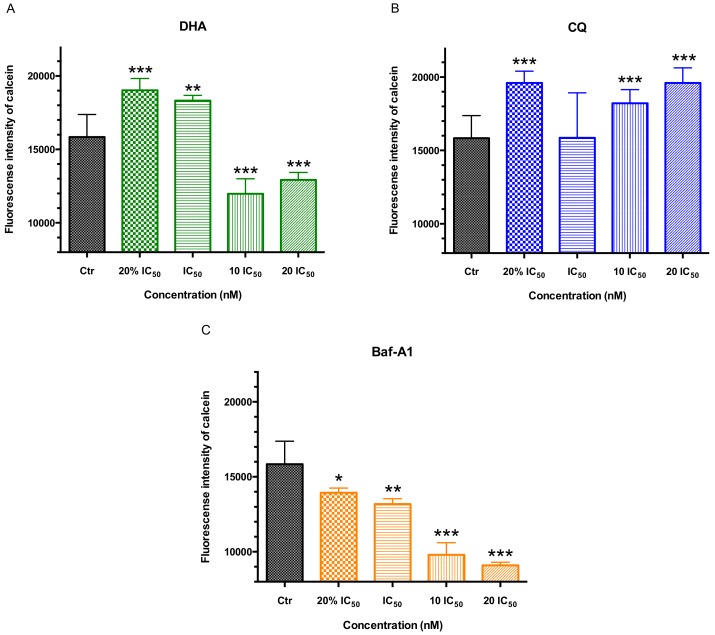
The fluorescence intensity of intracellular irons at trophozoite stage *P. f* 3D7 staining with calcein, AM after 2 h of single drug treatment with a series dose of antimalarial drugs DHA/CQ or V-ATPase inhibitor Baf-A1 by flow cytometry. (**A**) Fluorescence intensity of BCECF in trophozoite stage *P. f* 3D7 treated by DHA; (**B**) Fluorescence intensity of BCECF in trophozoite stage *P. f* 3D7 treated by CQ; (**C**) Fluorescence intensity of BCECF in trophozoite stage *P. f* 3D7 treated by Baf-A1. Ctr means control. Data shown are mean ± SEM (n ≥ 3). * *p* < 0.05, ** *p* < 0.01 or *** *p* < 0.001 vs. control.

The fluorescence intensity of intracellular irons staining with calcein, AM after 2 h of single drug treatment with a series dose of antimalarial drugs DHA/CQ or V-ATPase inhibitor Baf-A1 by flow cytometry was shown in Figure 6. Calcein, AM labeled the cytoplasmic LIP of the malaria parasite and low FI represented the higher content of ferrous irons. Seen from Figure 6A, the FI of calcein was decreased by DHA in low doses (20% IC_50_/IC_50_) and increased by DHA in high doses (10 IC_50_/20 IC_50_) significantly; also increased by CQ in low or high doses (20% IC_50_/10 IC_50_/20 IC_50_) groups. All doses of V-ATPase inhibitor Baf-A1 decreased the fluorescence intensity of calcein significantly, with a dose-effect relationship compared with control group.

#### 2.3.2. Combination Drug Treatment Testing

**Figure 7:**
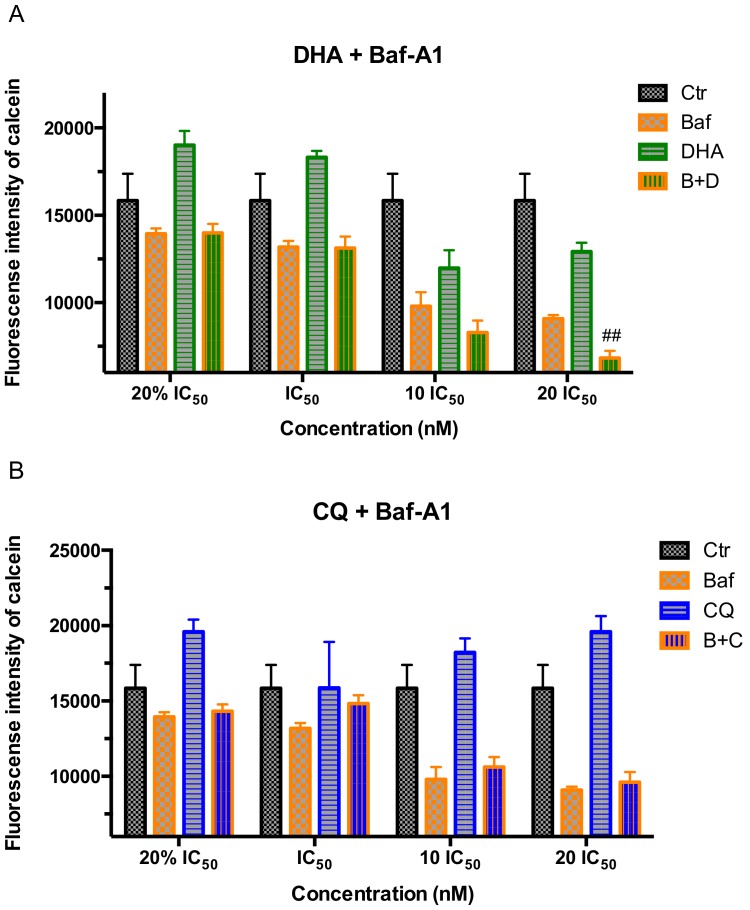
The fluorescence intensity of parasites in trophozoite stage *P. f* 3D7 staining with pH probe Calcein, AM after 2 h of combination drug treatment with a series dose of antimalarial drugs DHA/CQ and V-ATPase inhibitor Baf-A1 by flow cytometry. (**A**) Fluorescence intensity of BCECF in trophozoite stage *P. f* 3D7 treated by DHA combined with Baf-A1; (**B**) Fluorescence intensity of BCECF in trophozoite stage *P. f* 3D7 treated by CQ combined with Baf-A1. Ctr means control, B+D means DHA combined with Baf-A1, B+C means CQ combined with Baf-A1. Data shown are mean ± SEM (n ≥ 3). ## *p* < 0.01 vs. Baf-A1.

The fluorescence intensity of intracellular irons staining with calcein, AM after 2 h of combination drug treatment with a series dose of antimalarial drugs DHA/CQ and V-ATPase inhibitor Baf-A1 by flow cytometry was shown in Figure 7. High dose of DHA combined with Baf-A1 (20 IC_50_) showed a significant decreased effect (*p* < 0.01) on the FI of calcein, which represented the higher content of ferrous irons compared with single treatment by Baf-A1 group.

### 2.4. Gene Expression of V-ATPases in the Membrane of Parasite DV

**Figure 8:**
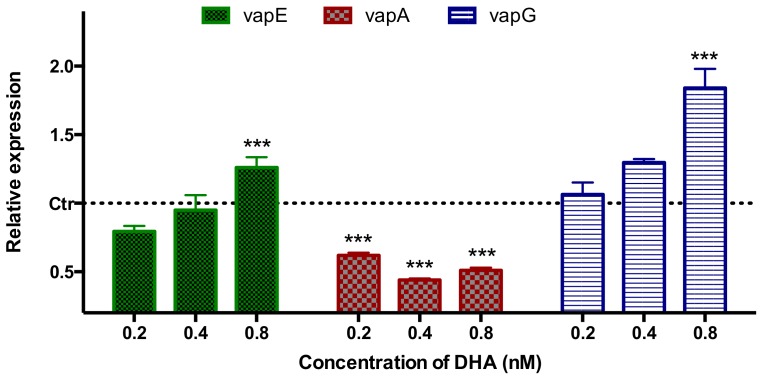
Gene expression of V-ATPases located in *P. f* 3D7 DV membrane at trophozoite stage by RT-qPCR. Ctr means control. Values are expressed as mean ± SEM of three independent experiments. *** *p* < 0.001 vs. control.

Gene expression was measured to generate the macromolecular machinery of drug DHA. Levels of mRNA abundance was quantitatively measured by RT-qPCR. Refer to PlasmoDB database, a genome database for the *Plasmodium* genus, 12 V-type proton ATPase subunits were found and only 3 V-ATPase subunits were reported to be located in the membrane of DV, including vapE, vapA, vapG. Gene expression levels of these V-ATPases related to DV of trophozoite stage *P. falciparum* strain 3D7 were tested, Glyceraldehyde-3-phosphate dehydrogenase (GAPDH) gene was served as the internal reference, relative gene expression was showed in Figure 8. Results showed that VapA was inhibited when parasites were treated with all low doses of DHA (0.2/0.4/0.8 nM) significantly (*p* < 0.001) compared with control. The expression of vapE and vapG was increased by 0.8 nM DHA with a significance (*p* < 0.001).

## 3. Discussion

IC_50_, the half maximal inhibitory concentration, represents the concentration of an inhibitor that is required for 50% inhibition of things like an enzyme, a cell, a cell receptor or a microorganism. It’s commonly used as a measure of drug effectiveness. In vitro results of drug treatment to test IC_50_ showed that *P. f* 3D7 was all inhibited by antimalarial drugs DHA/CQ and V-ATPase inhibitor Baf-A1 in a nanomolar concentration and the IC_50_ value of Baf-A1 was approximate to that of CQ. The result demonstrated that the V-ATPases is vital for *P. falciparum* parasite growth during the intra-erythrocytic stage.

In 1981, the concept of “Combination Index” was introduced, which includes the combination index (CI) equation and Fa-CI plot [20,21,22,23]. By dividing the equations in the presence and absence of inhibitor and by using the method of mathematical induction and deduction, the “median-effect equation” was derived by Chou [21,22]. The combination index method is based on that described by Chou and Talalay [22,23] and the computer software of Chou and Chou [23,24] and CalcuSyn. The ranges of CI and the symbols are refined from those described earlier by Chou [23]. CI < 1, =1, and >1 indicate synergism, additive and antagonism effect, respectively [22,23]. In this study, we provide evidence for antimalarial drug combinations, which DHA combined with Baf-A1 in a concentration of IC_50_ showed a synergism inhibitory effect on the growth of parasite (CI = 0.524), while it’s still needed to do more research in clinical trials for this result is proved only by in vitro experiments. However, considering malaria is a blood system infectious disease, the way of drug directly added to the culture dishes is similar to the clinical mode of administration in clinical tests, so infected RBCs or *Plasmodium* parasites which are cultured in vitro can be used to evaluate the drug effects of great reference value, support the results of drug combination use and explore the mechanisms of drugs DHA/CQ effect on parasites.

The DV of the *P. falciparum* maintains a low acidic lumen by means of the proton pump and V-ATPase. With the acidic pH optimum conditions, dozens of hydrolytic enzymes in the DV are activated. The acidic pH condition of the DV is thought to play a key role in the Hb digestion process to accumulate heme or ferrous irons within this organelle [25]. The pH homeostasis within the malaria parasites is conduced by proton transport, whose mechanism is involved in three targets in parasites: (1) proton pump, possibly P-type ATPase, which is inhibited by N,N9-dicyclohexylcarbodiimide or vanadate; (2) Na^+^/H^+^ antiporter, which is inhibited by amiloride and its analogue, EIPA; (3) V-ATPase, which is inhibited by bafilomycin A1 and concanamycin B [26]. Researchers have found that malaria parasite maintaining an intracellular pH homeostasis mainly involves V-ATPases within the DV and cytoplasma [27]. A V-ATPase-evoked acidic environment has been realized to be important for various biological phenomena including the transport of nutrients, ions, toxins, and organellar fusion events [26]. Aiming to develop new drugs against *P. falciparum* strains and considering that Hb digestion within the parasite DV is an important target, we tested the antimalarial activity of Baf-A1, which proved to be a highly valid inhibitor of malaria parasite growth with an IC_50_ value of 25 nM (Table 1). Low nanomolar concentration is required against the growth of *P. f* 3D7, which means V-ATPases are vital for parasite growth or DV.

The maintenance of pH homeostasis is critical for a variety of cellular metabolic processes. There are numerous existing methods for the measurement of intracellular pH by different probes or indicators. BCECF, AM, the most well-known intracellular ratiometric pH sensitive indicator, diffuses into cells, where it is cleaved by intracellular esterases to the unesterified form, which emits fluorescence according to the intracellular pH (pHi) [28]. BCECF, AM is wildly used in varies of cells and applied for the determination of intracellular pH in the form of single peak emission [29]. The FI of BCECF detected from parasite cultures demonstrates the intracellular pH or cytoplasma protons. Since IC_50_ experiments conducted above were assessed in 72 h, during the drug treatment on parasite cultures in 2 h, we increased the concentration level up to 10 IC_50_ values for obvious effects. Results (Figure 4) showed that Baf-A1, an inhibitor of V-ATPase, decreased the FI of BCECF and the parasite cytoplasmic pH, which is conduced by the inhibitory effect on transmembrane protons excreted across the plasma membrane and pumped into DV, a result of pH acidizing in parasite cytoplasma. Similar results [26], that Baf-A1 and concanamycin B had a decreased effect on the cytoplasmic pH of parasitized erythrocytes or infecting malaria cells, have been reported by Japanese scientist Mitsuko Hayashi and his colleagues.

Meanwhile, 0.2 IC_50_ DHA increased the pH of parasite cytoplasma, which showed opposite effects compared with Baf-A1, DHA treatment showed dose-effect relationship on parasite cytoplasmic pH. This result matched the results, showed above, that low concentration groups of DHA/CQ (0.2 IC_50_~0.8 IC_50_) combined with Baf-A1 had antagonism inhibitory effects (Table 1) on parasite growth. Low concentration groups of DHA/CQ combined with Baf-A1 showed antagonism effects that may induced by competitive inhibition with the V-ATPases target, which means DHA developed its antimalarial mechanism by the inhibition of V-ATPases to alkalize the parasite cytoplasm, while CQ might have no effect on V-ATPases. High concentration level of DHA (IC_50_ value) combined with Baf-A1 had a synergism inhibitory effect, which means DHA increased the protons in cytosol and by the same time, decreased the protons in the DV pumping by V-ATPases. On the other hand, Baf-A1 combination with DHA in a relative high concentration of IC_50_ showed a synergism effect, based on this preliminary result, it is believed that the combination of the artemisinin derivatives combined with Baf-A1 can be a hopefully legitimate choice for an alternative antimalarial combination therapies development.

It is reported that *P. falciparum* consumes up to 70–80% of the host cell Hb [30] but uses only up to 16% of the produced AAs for de novo protein synthesis [31]. During the digestion process of Hb, high concentrations of heme or ferrous irons are accumulated within the DV and parasites have to utilize and detoxify heme by informing Hz. Thus, heme metabolism is vital for malaria parasite biology. Most of the ferrous irons are formed from heme and the content of ferrous irons or hemes in DV is several times of magnitude higher than that of cytoplasm. Heme or ferrous iron homeostasis are crucial for intra-erythrocytic stage of *P. falciparum*. The mechanism of maintaining iron homeostasis or intra-erythrocytic labile iron pool involved in many pathways, including malaria parasite sensing, acquiring, utilizing, regulating, transporting and storing irons during the life cycle of the erythrocytic stage parasite.

Calcein, AM—a cell-permeable, non-fluorescent compound for monitoring cell viability, chemotaxis, cell adhesion and multidrug resistance—can be hydrolyzed by intracellular esterases to become fluorescent calcein in living cells. The acetoxymethyl ester of calcein is also used to detect drug interactions with multidrug resistance proteins (ABC transporters ATP-binding cassette transporter genes) in intact cells as it is an excellent substrate of the multidrug resistance transporter 1 (MDR1), P-glycoprotein and the multidrug resistance-associated protein (MRP1) [31]. The calcein, AM assay can be used as a model for drug-drug interactions, for screening transporter substrates and/or inhibitors; and to determine drug resistance of cells in vitro, including samples from patients [32]. Calcein green fluorescence is quenched by the stoichiometric binding of iron (1:1), leading to a decrease in the FI signal [33].

It’s reported that the intra-erythrocytic labile iron pool amounts to 20 mM iron, partitioned into Hb, ferritin, and the cytoplasmic labile iron pool [34]. Calcein, AM has been widely used to examine the cytoplasmic LIP of mammalian cells in these years. Calcein, AM is non-fluorescent, non-iron binding, neutrally charged, and easily permeates cell membranes. Upon cellular entry, intracellular esterases cleave calcein, AM into the green-fluorescent molecule calcein, which is then trapped within the cell. Calcein fluorescence is quenched by 1:1 stoichiometric binding of iron in pH range of 7–7.5 [35]. The addition of non-fluorescent, high affinity iron chelators removes iron from calcein and consequently increases calcein fluorescence, providing an effective method for assessing the labile iron content of cells. Calcein, AM has been utilized to assess the LIP of the heterogeneous cell populations of peripheral blood and bone marrow by FCM [36]. We tested the cytoplasmic LIP of parasite according to the methods of Martha Clark and Nancy C. Fisher [34]. Their application of the flow cytometry based Caclein, AM LIP assay has revealed that the LIP content of infected RBCs steadily increases with increasing maturation of the intra-erythrocytic stage of the parasite [37]. Intracellular labile iron pool probes are used to quantify the variation of ferrous irons. FI demonstrates the ferrous irons level of cytosol. While losing their membrane selectivity, iRBC allow ions (e.g., Na^+^, K^+^, Zn^2+^, Fe^2+^ and Ca^2+^), polar molecules (e.g., amino acids, glucose, purine nucleosides) and even antimalarial drugs (e.g., mefloquine, chloroquine) to pass into the cells readily [38]. Thus, we tested the ferrous irons variation by FCM using probe calcein, AM labeling the LIP mainly related to parasite cytosol. Cytoplasmic LIP, seen from the results (Figure 6), was decreased by Baf-A1, a single treatment with high concentration of DHA (10 IC_50_), and a high concentration dose of DHA (20 IC_50_) combined treatment with Baf-A1 showed a synergism effect on increasing ferrous irons in cytosol. The inhibitory effect caused by Baf-A1 cued the fact that cytoplasmic protons increased with ferrous irons increasing to balance the ions homeostasis. The results of DHA on LIP demonstrated that DHA caused the proton increasing and ferrous irons decreasing in cytosol, which caused the toxic heme or ferrous irons accumulated in DV increasing and induced the death of parasite.

The in vitro experiments provided evidence that the mechanism of antimalarial drugs DHA may be related to interrupting pH or ferrous homeostasis and heme accumulating in DV, which is different from CQ. Scholl and coworkers support the evidence that malaria parasites utilize labile bioavailable iron pool(s) in RBC cytoplasm rather than haemozoin iron in the DV in order to synthesize their own heme in the mitochondria and apicomplast, while iron chelators can compete the iron utilization process and kill the parasite based on iron deprivation [39]. Further studies should be designed to detect the mounts of heme or ferrous irons within DV by extract.

DV is also the vital organelle responsible for these iron pathways in parasite. Our work showed that the antimalarial or multidrug resistant mechanisms of artemisins and quinolones are related to protons and irons homeostasis in parasites. Plasma membrane V-ATPases are responsible for active extrusion of protons from the parasite cells, while V-ATPases located in DV membrane are responsible for protons pumping into parasite DV. To investigate the effect of DHA on the malaria parasite, we tested in a low concentration which could not lead to the death of parasites but have an effect on the parasites and verified by RT-qPCR to measure the relative gene expression of V-ATPases, located in the membrane of DV, results showed that DHA decreased the expression of vapA significantly. Although this technique is still used to assess gene expression, it requires relatively large amounts of RNA and provides only qualitative or semi quantitative information of mRNA expression levels [40]. Further studies should be performed on V-ATPase proteins to evaluate a vast array of functions within this organelle and antimalarial drugs.

## 4. Materials and Methods

### 4.1. Materials

DHA was provided by researcher Yang Lan of China Academy of Traditional Chinese Medical Sciences. CQ, Baf-A1, D-sorbitol and gentamycin sulfate were purchased from BioRuler (Danbury, CT, USA). SYBR Green I nucleic acid gel stain (Invitrogen 10,000 × concentrate in DMSO), labile iron pool probe calcein, AM and SYTO^TM^ 61 red fluorescent nucleic acid stain were purchased from Invitrogen by Thermo Fisher Scientific (Eugene, OR, USA). Tris-HCL, EDTA, saponin, Triton X-100, hypoxanthine, dimethyl sulfoxide (DMSO) and HEPES were purchased from Sigma Life Science (Spruce Street, St. Louis, MO, USA). RPMI Medium 1640, AlbuMAX II and L-glutamine were purchased from Gibco by Life Technologies (Grand Island, NY, USA). NaHCO_3_ and D-Glucose were purchased from Sinopharm Chemical Reagent (Beijing, China). RT-qPCR primers were purchased from Invitrogen Trading (Shanghai, China). RNAsimple Total RNA Kit, FastKing RT Kit (With gDNase) and Talent qPCR PreMix (SYBR Green) were obtained from TIANGEN BIOTECH (Beijing, China). pH probe BCECF, AM was purchased from KeyGEN BioTECH (Nanjing, China). Suspended leukocyte-reduced red blood cells (sickling negative; O rhesus positive) were taken from Red Cross Society of China (Beijing, China).

*Plasmodium falciparum* laboratory strain 3D7 was maintained long-term preservation by liquid nitrogen and subcultured by our lab.

### 4.2. Methods

#### 4.2.1. Drug Solutions

The malarial culture medium (MCM) consisted of filter sterilized RPMI 1640 solution supplemented with HEPES (0.6%; *w*/*v*), AlbuMAX II (0.5%; *w*/*v*), glucose (0.2%; *w*/*v*), L-glutamine (0.02%; *w*/*v*), hypoxanthine (0.025 mg/mL), gentamicin (0.025 mg/mL) and buffered with NaHCO_3_ (0.2%; *w*/*v*) added to the final solution.

Incomplete medium (UB) was prepared almost the same as MCM excluding AlbuMAX II.

Malaria 10 × Lysis buffer solution, consisted of 1 M Tris-HCl (30%; *v*/*v*; pH 7.5), 0.5 M EDTA (15%; *v*/*v*; pH 8.0), saponin (0.12%; *w*/*v*), and Triton X-100 (1.2%; *v*/*v*), was prepared in advance and stored at 4 °C.

Five mM DHA, CQ and Baf-A1 stock solutions were all dissolved in DMSO and sterilized by passing through filter membranes (Millipore 0.22 μm). Working solutions were serially diluted in MCM/UB to achieve the final concentrations ranging from μM to nM. Stock solutions were stored at −80 °C and work solutions were stored at −20 °C. All solutions were thawed before use.

#### 4.2.2. *Plasmodium falciparum* Culture In Vitro

The erythrocytic stage of *P. falciparum* strain 3D7 was routinely cultured in vitro based on the method described by Trager and Jensen [41] with minor modifications. The parasites were cultured in erythrocytes fortified in MCM. The parasites were grown in culture dishes incubated under a gas phase of 90% N_2_, 5% CO_2_ and 5% O_2_ at 37 °C. Parasites were mostly synchronized at ring stage with 5% D-sorbitol. Infected RBC (iRBC) suspension was spun down at 1900 rpm, room temperature for 3 min and the supernatant was discarded. The cell pellet was resuspended in 10 times volume of sterile 5% D-sorbitol (*w*/*v*) solution and incubated at 37 °C for 10–15 min. After centrifugation, cell pellets containing both intraeythrocytic ring and early trophozoite stage parasites were resuspended in fresh MCM and haematocrit of the cultures was kept in 2%. The cultures were maintained daily by changing the media. Parasite viability and growth were monitored by light microscopy. Parasitaemia (P/%) levels in the cultures were kept between 2–9% by adding fresh RBCs. RBCs were washed three times by RPMI 1640 and centrifuged at 1900 rpm at room temperature for 10 min before use.

#### 4.2.3. Single Drug Treatment to Test IC_50_

Malaria parasites at the ring stage were synchronized for at least three times before the experiment. After synchronization, *P. falciparum* strain 3D7 at early ring stage (less than 6 h) were resuspended in new MCM to obtain 4% haematocrit and parasitaemia level was adjusted to 1% by adding fresh RBCs. Stock solutions of DHA/CQ/Baf-A1 were diluted by MCM to a series of various concentration solutions as single drug working solutions with DMSO% < 0.5% (*v*/*v*) and were dispensed in triplicate test wells. RBCs/iRBCs suspension (100 μL) was transferred to each well of 96 culture plate. RBCs/iRBCs with no drug treatment were plated as comparison of 0%/100%. Plate was blended for 10 min to fully mix seeded RBCs/iRBCs with drug solutions. After 72 h incubation, parasites at trophozoite stage were above 90%. The supernatant of each well (110 μL) were abandoned. Freshly prepared malaria 10 × lysis buffer solution with 200-fold dilution of 10,000 × SYBR Green I (10 μL) was added to each well, thoroughly mixed until complete haemolysis and incubated in the dark at room temperature for 1 h. Fluorescent intensity (FI) was measured by a Multiscan Spectrum (SpectraMax i3x, Molecular devices) at the wavelengths of Ex = 535 nm and Em = 485 nm. Half maximal inhibitory concentration (IC_50_) value of each drug was determined by the dose response curve representing used drug concentrations on the X axis and parasitaemia (%) on the Y axis. IC_50_ value represents the concentration of a compound where 50% of its maximal inhibitory effect is observed and it is commonly used as a measure of drug’s potency.

#### 4.2.4. Antimalarial Drug Combination Treatment with Baf-A1

After IC_50_ measurement of DHA/CQ/Baf-A1, DHA/CQ combined treatment with Baf-A1 were almost conducted by the same method with single drug treatment to test IC_50_, except tested combination drug treatment solutions were diluted to serious concentration solutions of the 80%, 60%, 20% IC_50_ value and two combined drugs with same ration of solutions were added at the same well of 96 culture plate. Single drug treatment with concentrations of 80%, 60%, 20% of the IC_50_ value was plated as comparison to test the synergism or antagonism effect of drug combination treatment.

#### 4.2.5. Cellular pH and Labile Iron Pool Measurement by Flow Cytometry Using Probes BCECF, AM/Calcein, AM

The double labelling method was used in this flow cytometry (FCM) approach, which allows for the analysis of the LIP of a mixed population of uninfected erythrocytes and erythrocytes infected by *P. f* 3D7.

The same part of double labelling method with different probes used in FCM was handled as following: iRBCs with parasite at trophozoite stage (30–38 h) above 90% were centrifuged at 1500 rpm, room temperature for 2 min, and resuspended in UB to obtain 1 μL/mL haematocrit. Parasiteamia was adjusted to 5–10%. iRBCs groups with different concentrations of drugs were all labeled with probes firstly, then incubated with different drugs at 37 °C for 2 h and detected by Beckman Coulter CytoFLEX (South Kraemer Boulevard Brea, CA, USA). A different part of double labelling method with different probes was as following:

For pH probe BCECF, AM testing: iRBCs groups were stained with BCECF, AM firstly, at the final concentration of 5 μM, water-bathed for 50 min at 37 °C, washed two times with UB. Then, iRBCs were stained with SYTO™ 61 red fluorescent nucleic acid stain at the final concentration of 0.5 μM to stain the nucleic acids of the malaria parasites, water-bathed for 15 min at 37 °C, washed two times with UB. Finally, the FI signals (wavelength of Ex = 488 nm/Em = 525 nm for calcein, AM; wavelength of Ex = 625 nm/Em = 645 nm for SYTO™ 61) of the cells were analysed through flow cytometer.

For labile iron pool probe calcein, AM: almost the same method as used above, except the first label was stained with calcein, AM at a final concentration of 0.125 μM and water-bathed for 15 min at 37 °C, the FI signals (wavelength of Ex = 488 nm/Em = 535 nm for BCECF, AM; wavelength of Ex = 625 nm/Em = 645 nm for SYTO™ 61) of the cells were analysed through flow cytometer.

#### 4.2.6. Measurement of Gene Expression of V-ATPases

Different doses of DHA: (0.2 nM, 0.4 nM, 0.8 nM) were added to iRBCs with parasites at trophozoite (32–36 h) stage above 90% and cultured in vitro, after incubation for 2 h at 37 °C, iRBCs were pyrolysised by saponin (0.1%; *w*/*v*), parasites were washed with 1 × PBS for two times to remove RBC contamination, total RNA of the parasites was extracted from the bottom sedimentation with RNAsimple Total RNA Kit in accordance to instruction book. cDNA was generated using FastKing RT Kit (With gDNase) by reverse transcription in accordance to instruction book. Glyceraldehyde-3-phosphate dehydrogenase (GAPDH) gene was served as the internal reference, RT-qPCR was conducted on cDNA samples with the following primer sequences, according to Table 4, using Talent qPCR PreMix (SYBR Green) in accordance to the instruction book. GAPDH was tested as internal control for relative gene expression.

#### 4.2.7. Statistical Analysis

Data for studies were presented as mean ± SEM with from the results of experiments conducted at least three times. The GraphPad Prism one-way ANOVA (GraphPad Software Ver. 6, San Diego, CA, USA) was used to establish any significant differences among the means of groups.

## 5. Conclusions

In conclusion, our findings suggest that DHA may prevent parasite growth by interferencing with the ferrous iron hemostasis, inhibiting V-ATPases or proton pumping, and alkalizing the DV. In addition, DHA had an inhibitory effect on proton transport and the homeostasis of intracellular pH. DHA inhibited the cytoplasmic labile iron pool in combination of V-ATPases inhibitor Baf-A1 with a synergism effect that proved to be the irons activated mechanism of DHA. Our preliminary results measured by in vitro experiments shown that DHA combined with V-ATPases inhibitors provides a (hopefully) new combination drug target for antimalarial combination therapies. Further in vivo research should and will be conducted in vivo to confirm the target. CQ showed an effect on the proton hemostasis or acidic pH environment within DV by inhibiting the growth of parasites and had only an antagonism effect or nearly additive effect on *Plasmodium falciparum* when combined with Baf-A1. The targeted site of gene expression of V-ATPases were explored, it is verified that V-ATPases located in the membrane of DV may be the targets to develop the inhibitory effect of DHA on *Plasmodium falciparum*.

## Figures and Tables

**Table 1:** The targets of antimalarial drugs DHA/CQ and V-ATPase inhibitor Baf-A1.

Drugs	Targets
CQ	Accumulation inside the DV; interference with heme; interference with Hz; effect on the membrane integrity; inhibition of lysosomal proteins; interference pH
DHA	Activation with ferrous irons; accumulation of ROS; interference the Hb digestion
Baf-A1	Inhibition of V-ATPases; influence on proton pump; inhibition of autophagy; inhibitory activity against bacteria and tumour cell lines etc.

**Table 2:** IC_50_ values of DHA/CQ/Baf-A1 against *P. f* 3D7 in vitro.

Drug	Mean IC_50_ ± SEM (nM)
DHA	4.12 ± 0.53
CQ	18.74 ± 0.56
Baf-A1	25.15 ± 3.39

Values are expressed as mean ± SEM of three independent experiments.

**Table 3:** Combination index values and effects of DHA/CQ with Baf-A1 against *P. f* 3D7 in vitro.

Drug Combination	CI for Experimental Values				
	20% IC_50_	40% IC_50_	60% IC_50_	80% IC_50_	IC_50_
DHA + Baf-A1	4.954	2.229	1.584	1.02	0.524
Combination effect	−−−−	−−−	−−−	±	+++
CQ + Baf-A1	2.160	2.678	2.042	1.549	1.012
Combination effect	−−−	−−−	−−−	−−−	±

CI < 1, =1, and >1 indicates synergism (+), additive effect (±), and antagonism (−), respectively. (Range of 0.3 < CI < 0.7 means synergism, 0.9 < CI < 1.1 means nearly additive, 1.45 < CI < 3.3 means antagonism, 3.3 < CI < 10 means strong antagonism effect).

**Table 4:** Primer sequences of genes tested by RT-qPCR.

Gene Name	Gene ID of PlasmoDB	Primer Sequences (5′-3′)	
GAPDH	PF3D7_1462800	F	CATGTGAGGTAACCCACGCT
		R	CTTTGGTGGGGCGGACATAA
vapE	PF3D7_0934500	F	TACCTCCACCACCTACACCTG
		R	GATGGCTAGTTTGAGACGCAC
vapA	PF3D7_1311900	F	GGCCTGTTCGTGATCCTAGAC
		R	TCCACAACCAAATGCACCAG
vapG	PF3D7_1323200	F	AGGATGTGAGGGCAAAGATGT
		R	ACAGCTTCGTCTTCTGCTG

F means forward sequence and R means reverse sequence.
